# Optimization of the Casualties’ Treatment Process: Blended Military Experiment

**DOI:** 10.3390/e22060706

**Published:** 2020-06-25

**Authors:** Jan Hodický, Dalibor Procházka, Roman Jersák, Petr Stodola, Jan Drozd

**Affiliations:** 1 NATO Headquarters Supreme Allied Commander Transformation, Norfolk, VA 23551, USA; jan.hodicky@act.nato.int; 2 Centre for Security and Military Strategic Studies, University of Defence, 66210 Brno, Czech Republic; dalibor.prochazka@unob.cz; 3 Center of Simulation and Training Technologies, 60200 Brno, Czech Republic; roman.jersak@unob.cz; 4 Department of Intelligence Support, University of Defence, 66210 Brno, Czech Republic; 5 Department of Tactics, University of Defence, 66210 Brno, Czech Republic; jan.drozd@unob.cz

**Keywords:** complex systems, system dynamics, casualty treatment optimization

## Abstract

At the battalion level, NATO ROLE1 medical treatment command focuses on the provision of primary health care being the very first physician and higher medical equipment intervention for casualty treatments. ROLE1 has paramount importance in casualty reductions, representing a complex system in current operations. This study deals with an experiment on the optimization of ROLE1 according to the key parameters of the numbers of physicians, the number of ambulances and the distance between ROLE1 and the current battlefield. The very first step in this study is to design and implement a model of current battlefield casualties. The model uses friction data generated from an already executed computer assisted exercise (CAX) while employing a constructive simulation to produce offense and defense scenarios on the flow of casualties. The next step in the study is to design and implement a model representing the transportation to ROLE1, its structure and behavior. The deterministic model of ROLE1, employing a system dynamics simulation paradigm, uses the previously generated casualty flows as the inputs representing human decision-making processes through the recorder CAX events. A factorial experimental design for the ROLE1 model revealed the recommended variants of the ROLE1 structure for both offensive and defensive operations. The overall recommendation is for the internal structure of ROLE1 to have three ambulances and three physicians for any kind of current operation and any distance between ROLE1 and the current battlefield within the limit of 20 min. This study provides novelty in the methodology of casualty estimations involving human decision-making factors as well as the optimization of medical treatment processes through experimentation with the process model.

## 1. Introduction

Casualty reduction is the main objective of any planned mission. There are two main categories of reduction approaches. The first, the efficiency of medical treatment, is the main domain of this study. The second, the higher operational effectiveness of own forces, is beyond the scope of this paper.

Founded in military history, NATO nations military medical treatment is classified under five categories [1]. Each category defines an essential treatment capability it is able to provide. Medical treatment categories are referred to as Roles 0–4. Medical treatment capability differs from the first response carried out by every soldier, defined as Role 0, up to full hospital treatment capability in ROLE4.

**Figure 1:**
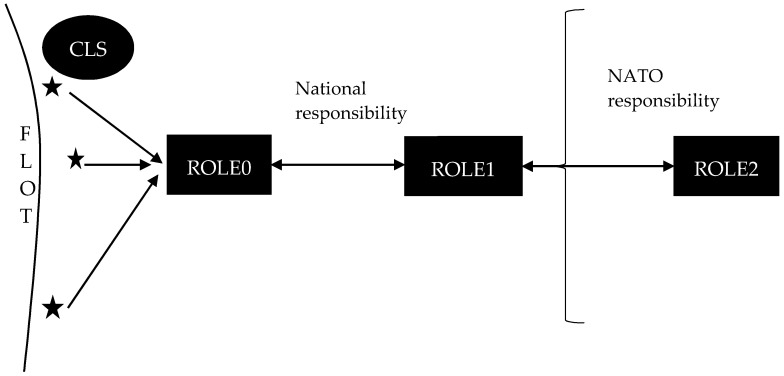
Military healthcare treatment in the area of operation from casualties’ occurrence up to ROLE2.

Figure 1 describes a simplified view of the healthcare treatment in the area of operation up to the ROLE2 functions. This structure, its variants and the actual primary operational environment in which it is executed, create a complex system.

Casualties occur in the area of responsibility of own forces, close to the forward line of own force (FLOT) identifying the forward location of covering and screening forces. Casualties are given immediate care by a combat life saver (CLS) military personal and they are transported into the ROLE0 place under the company commander command, while waiting for transport to ROLE1.

ROLE1 focuses on the provision of primary health care comprised of first aid, triage, resuscitation and stabilization. It is the very first physician and higher medical equipment intervention in the military medical treatment process. Therefore, ROLE1 has paramount importance in casualty reductions in the current as well as in future dynamic and complex operational environment [2].

Every casualty must go through ROLE1 treatment, as that is within the national responsibility environment. Therefore, nations deploying battalions under NATO command follow national recommendation for its organization and internal structure. There is a ROLE1 structure parameter recommendation from the NATO nations level [3]. However, these parameters are constructed from a statistical analysis of historical reviews of past battles, rather than reflecting the current and future dynamic operational environment.

ROLE1 is understood as well to be a mobile platform, therefore its position in respect to the current battlefield plays a crucial role in casualty reductions [4].

Within NATO operations, ROLE2 is not predefined as a national responsibility. It is characterized by its ability to perform all surgical interventions in addition to ROLE1 responsibility. The functions of ROLE3 and ROLE4 are very close to the full capacity of hospitals in the civilian domain and their functions are beyond the scope of the study.

The aim of the study is to optimize the parameters of the ROLE1 military field dressing station at the battalion level from the perspective of estimated casualties at the current battlefield as described in a complex system.

To reach the study objective, a model of the ROLE1 process is created and the inputs for the simulation of the ROLE1 model are experimentally gathered. Finally, the execution of a factorial experimental design helps understand the simulation run results and their relevance to the parameters of the ROLE1 structure.

Therefore, Section 2 contains literature reviews related to the experimentation as well as studies similar to the military field treatment. Section 3 formulates the optimization problem and parameters of ROLE1 structure and behavior. Section 4 contains the methodology of the whole experiment and the details of the deterministic model implemented, as founded in the previously executed computer-assisted exercise (CAX) involving the human factor in the decision-making processes. Section 5 discusses the results of the design of the experiment with the ROLE1 model. The conclusion demonstrates the study’s impact on theory and practice and explains the term “blended military experiment”.

The originality of the study is two-fold. It provides evidence-based estimates of parameters of the national structure of ROLE1 and it demonstrates a new methodology for military experimentation founded in reusing recorded flows of CAX events, to generate inputs for an experiment with a designed model to reduce the uncertainty coming from a complex environment, as well as from human influence in the decision-making processes.

## 2. Literature Review

The literature review is divided into two parts that form the primary building blocks of the study methodology. The first part of the literature review is related to the experimentation at ROLE1. The second part deals with the casualty estimations on the battlefield, as these are a prerequisite for the ROLE1 structure parameters’ optimization.

The objective of the experiment by Lynch et al. [5] was to establish the average treatment times in ROLE1 and ROLE2 and having done so, to evaluate whether the national facility is capable of meeting NATO objectives. A single-run medical field exercise was carried out with pre-scripted simulated casualties serving as the experiment inputs. In this way, the experiment does not optimize the structure parameters of ROLE1. ROLE1 was described by a single time-treatment parameter and the positional distances between ROLE0, ROLE1 and ROLE2 were not varied and therefore not taken into consideration.

Howard et al. [6] have a different approach: when a model of the entire treatment process was implemented, followed by the statistical analysis of simulated results to reveal if new features in the processes such as tourniquets, blood transfusions and prehospital transport, had influence upon decreased casualties in the battlefield, there was no field exercise; the model was built upon historical data from the battlefield. The ROLE1 structure sustaining the current and future operational environment requirements were not the objective of this study.

As for the second part of the literature review, there are, in principle, two approaches to casualty estimation. The first is focused on the statistical analysis of historical data. The second approach builds upon a simulation of combat operations.

Statistical analysis of historical data depends upon human factors because the decision makers were involved in the operations. It is not a case of closed-loop simulation where features of human behavior are simulated to limited extents and with limited validity. On the other hand, analyzing casualty estimate statistics alone is not sufficient either. They do not reflect the current and future operational environment. The simulation of combat operations can replicate an expected operational environment based on the most likely scenarios, however, human decisions must be replicated and validated. All in all, decision making can be viewed as a computational process that progressively eliminates alternatives, thereby reducing uncertainty, and therefore it needs to be reflected in the operational environment [7].

Studies using historical data gathered from the past battles are demonstrated in [8,9,10,11]. All the referenced statistics on casualties are related to the history of WW1–WW2 or to the very asymmetric military environment. Therefore, it cannot be replicable into the current and future predicted full-size operations, where forces with similar capabilities face each other. Moreover, it is not trivial to get the common denominators [12] from these statistics to be used further in the experimentation phase.

Sim at al. [13] demonstrate a simulation approach to casualty estimates employing the agent-based modeling and simulation paradigm. This solution introduces a theoretical model with the limitation of not involving human factors in the decision making process. Automata-making human decisions leads to the questioning of adequate validation processes.

Another method to gather estimates on casualties is applying the generalized recommendations on casualty estimates from NATO [4]. This approach is too general, with very low reality for the following experimentation, and cannot provide sufficient details to model a casualty flow in time.

It is evident that there is no methodology of casualty estimations employing the human decision factors and in parallel, reflecting the current and future operations environment.

## 3. Problem Formulation

Having previously explained that the functions of ROLE1 are a national responsibility, each nation must therefore design ROLE1 structure parameters to minimize life risk in the operation. Having ROLE1 structure parameters as the main objective of this study, the operational environment including human decisions must be taken into consideration. This creates a complex system, with nonlinear, dynamic and emergent behaviors, with the different types of information flow and causality in general stochastic processes, inferences and coupling structures and parameters of system dynamics [14]. As a consequence, the operational environment is defined by omnipresent uncertainty and a lack of control [15]. To deal with such complexity, both decomposition and abstract approaches must be employed.

**Figure 2:**
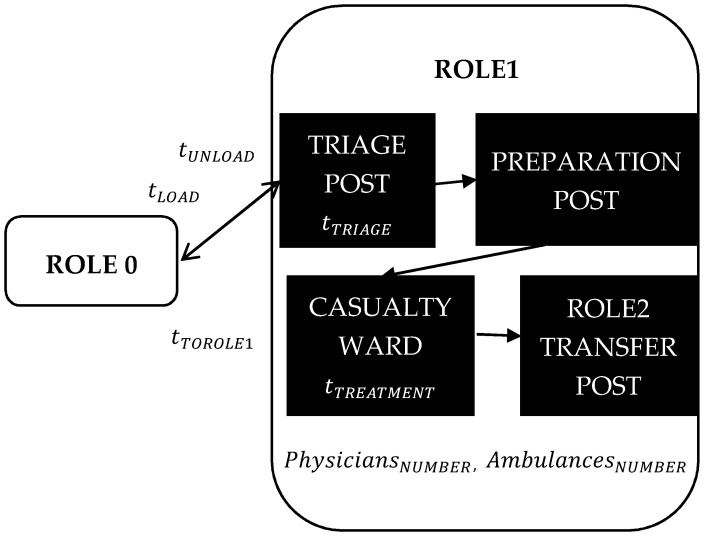
ROLE1 process described as the flow of its subprocesses characterized by the elapsed time of their function.

The operational health care system, up to and including ROLE1, is composed of two parts. The first part of the system represents battlefield casualties as its main output. The second part, represented by a structure of the ROLE1, consumes the outputs of the first part, trying to satisfy all casualty needs within two hours of the casualty appearance on the battlefield. The two hour limit given by NATO [1] must be fulfilled without exception. ROLE1 is described as the flow of the casualty treatment as it occurs in reality, see Figure 2. As with any other flow, the subprocesses in the flow are characterized by the elapsed time of their function.

Casualties are brought from ROLE0 to ROLE1 by the ambulance waiting in ROLE1, for transportation orders from the battalion commander. The loading time per casualty ($t_{LOAD}$) expresses the delay of the ambulance while loading the casualty in ROLE0. The unloading time per casualty ($t_{UNLOAD}$) expresses the delay of the ambulance while unloading the casualty in ROLE1. These parameters will influence the ambulance availability, because it waits until being fully loaded or unloaded. The distance between ROLE0 and ROLE1 is expressed as the total time needed to come to ROLE0 from ROLE1, to put a casualty into an ambulance and bring them to ROLE1 ($t_{TOROLE1}$). This transportation process is further modelled and described in Section 4.2.

In the triage post, casualties are classified into three casualty categories. The category P3 equals light wounded, P2 equals medium wounded and P1 equals seriously wounded casualties. After that, the casualty is moved to the preparation post, where they are prepared by the following surgery at the casualty ward. After surgery, they are moved to the ROLE2 transfer post for transport for further treatment. ROLE1 functionality and structure parameters are abstracted into the times of triage ($t_{TRIAGE}$), treatment ($t_{TREATMENT}$), the total number of physicians ($Physicians_{NUMBER}$) and ambulances ($Ambulances_{NUMBER}$). This casualties’ treatment at ROLE1 is further modelled and described in Section 4.2.

Having described the ROLE1 structure parameters and the method by which operational medical treatment is executed up to ROLE2, the optimization problem is formalized as follows: finding variants of ROLE1 structure parameters that satisfy the requirement of having a maximum of 2 h between the casualty’s introduction and the moment when a patient is ready for transportation to ROLE2.

## 4. Methodology

To obtain insights on the optimization problem, two interrelated models of a reality were proposed to cover the whole process, starting from the casualty being introduced into the battlefield up to the moment of its transportation to ROLE2. The first model represents casualties in the battlefield and the second one represents the transportation to ROLE1, as well as the behavior and events flow of ROLE1.

### 4.1. Model of Battlefield Casualties

The operational environment is a complex system that has plenty of nodes with non-linear and unknowable emergent behavior. Nodes, like a single soldier, battalion, brigade or non-governmental organization, are represented by entities with different levels of granularity, mutual communication and self-synchronization. Friction among these entities creates casualties. It is not possible to run the field experiment with actual entities to generate casualties; therefore, other means must be used to create a model, as described in the literature review.

**Figure 3:**
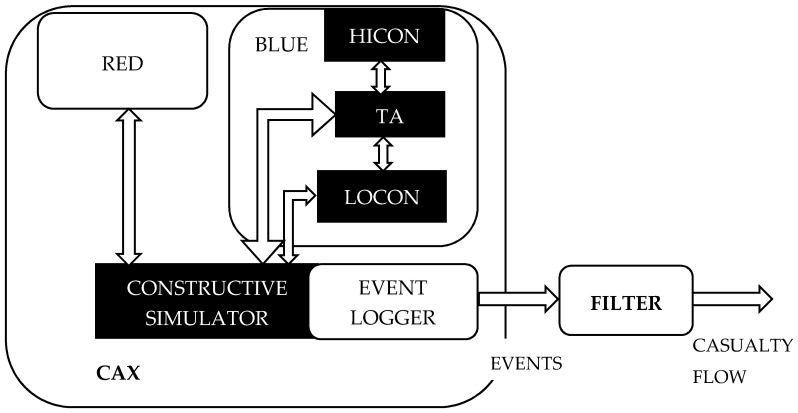
Computer assisted exercise (CAX) architecture composed of a constructive simulation, red force (RED) and blue force (BLUE) coupled with a transformation mechanism generating the casualty flow.

A design of the battlefield casualties model in the study is founded upon the idea to reuse the friction data generated from the already executed computer assisted exercise (CAX), while employing a constructive simulation. Figure 3 describes the CAX architecture in a simplified view as a composition of three basics elements.

The first one, constructive simulation, contains simulated human decision-making and a simulated environment with simulated effects. Therefore, units with defined levels of resolution are replicated, their behavior is encoded in the simulator and human beings are usually given ordinary tasks: move to, defend the position, speed up, etc. The level of detail in actions depends on the resolution of a constructive simulation [16].

The second element, red force (RED), represents all the activities and behaviors of enemy forces. Usually, experienced officers play this role and create inputs for a constructive simulation that replicates enemy units.

The last element, blue force (BLUE) represents all the activities of own forces. It is divided into three main groups: higher control (HICON), lower control (LOCON) and training audience (TA). TA is usually the commander’s staff who is trained to reach defined CAX training objectives. TA gives orders to LOCON to execute these orders in the constructive simulation. LOCON gathers data from the constructive simulation and informs the TA about the current situation and by doing so, creates situational awareness. HICON is used to represent a superior command level to TA, specifying a desired end state and supporting situational awareness from their perspective.

Any constructive simulation contains an event logger (EL) that saves all the events and messages occurring during the CAX execution. The main purpose for doing so is to enable an after action review (AAR), where the TA is confronted with their activities and the causes and effects of related activities in the simulated battlefield are explained. Therefore, EL can be used to replay, as many times as needed, all the events ordered in the proper time frame, perfectly mimicking the flow of events driven by human decisions. The level of detail of the events being recorded depends on the architecture of the constructive simulation [16].

These events can be transformed in the filter into series of events that are related to the studied object; in this case, to those events related to casualties on the battlefield and generating required casualty flows in the time and geographic distribution of P1, P2 and P3 casualty types.

Reusing data recorded from CAX is a unique approach, because it adds a human decision dimension to the simulation by employing the training audience. It overcomes the drawbacks of current closed-loop simulations that express a lack of human decision models.

### 4.2. Model of ROLE1 Structure and Behavior

The ROLE1 structure and behavior model applies system dynamics. System dynamics is a rigorous modelling and simulation paradigm, enabling to model the formal representation of complex systems and to design more effective policies and organizations through computer simulation [17]. The presented model is based on a stock and flow diagram, where stocks represent accumulations and a system state and their values are changed by incoming or outcoming flows (inflows and outflows).

A casualty is transported from ROLE0 to ROLE1 before it can be treated there accordingly. Therefore, the model forms two parts. The first covers the transportation mechanism from ROLE0 to ROLE1 and the second one represents the casualty treatment in ROLE1.

**Figure 4:**
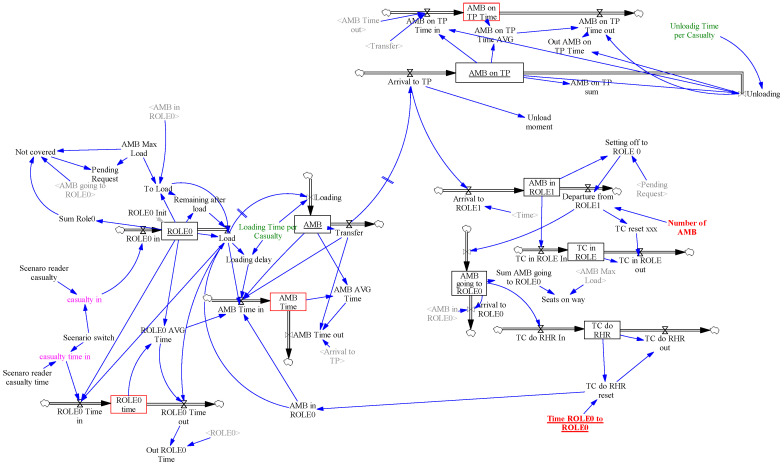
Transfer of the casualties from ROLE 0 to ROLE1 modelled in the system’s dynamics modelling and simulation paradigm.

The first part of the model (see Figure 4), represents the casualties’ transportation mechanism to ROLE1 in numbers, casualty types and time instances. The model of battlefield casualties, described in Section 4.1, creates the inputs for this part of the model.

**Figure 5:**
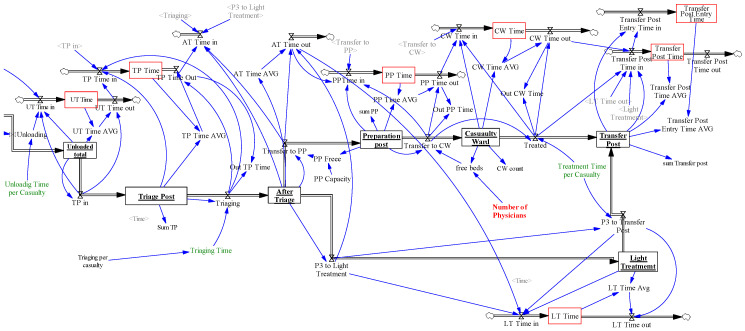
ROLE1 casualty treatment modelled in the system’s dynamics modelling and simulation paradigm.

*Scenario reader casualty* and *Scenario reader casualty time* are the variables generating casualty time series data from external files. Data are transformed according to the concrete scenario given by the parameter *Scenario switch* in inflows *RHR in* and *ROLE0 Time in*, respectively. The casualties are gathered in the *ROLE0* stock, loaded to the *AMB* stock (representing ambulances) through the *Load* and *Loading* flows and unloaded from *AMB on TP* stock on the *Triage Post* (see Figure 5) via the *Unloading* outflow. The transport from ROLE0 to the triage post is modelled by the *Transfer* outflow and the delay given by the variable *Time ROLE0 to ROLE1* = *Time ROLE1 to ROLE0* (hidden to simplify the figure). Delays caused by loading and unloading casualties are represented by *Loading Time per Casualty* and *Unloading Time per Casualty* parameters. The *ROLE0 Time*, *AMB Time* and *AMB on TP Time* stocks represent the co-flows to calculate the average time spent by casualties in corresponding stocks. These stocks are represented as red boxes in Figure 4.

All these stocks and flows are internally represented as three dimensional arrays, where each dimension corresponds to a casualty category (P1, P2, P3). When a casualty leaves a stock, it takes an average time amount from the corresponding time stock by its category and forwards it as a contribution to the next time stock.

The stocks *AMB in ROLE1* and *AMB going to ROLE0* represent the ambulances waiting in ROLE1 or on their way to ROLE0, which are represented by a six-dimensional array (maximal number of ambulances) where the *Number of AMB* parameter limits the ambulance availability in the simulation.

The casualties in *ROLE0* stock invoke a request for ambulances. The request is compared to ambulances already on the way to ROLE0 and a minimal number of available ambulances (*AMB in ROLE1*) covering the request sent to ROLE0. If the current request is satisfied, the *Pending Request* variable is set to 0, otherwise it is set to 1.

The loading process is triggered by the *AMB in the ROLE0* variable, as well as the *Loading Time per Casualty* variable which delays ambulance set off times, according to the number of casualties to load. Preference is given by casualty type, so P1 is preferred to P2 and P2 is preferred to P3.

The second part of the model (see Figure 5) represents the treatment of casualties in ROLE1. The casualty stocks are *Unloaded Total*, *After Triage*, *Preparation Post*, *Casualty Ward* and *Transfer Post*. Corresponding time co-flows are marked as red boxes. The function of the *Unloaded Total* stock is only to generate an integer number of casualties on the triage post. The *After Triage* stock represents triaged casualties. The *Transfer to PP* and *P3 to Light Treatment* outflows separate the casualties according to their types. P1 and P2 casualty types are required to be treated by Physicians in the Casualty Ward. The P3 casualties are treated separately by other staff than physicians, therefore it creates no extra load to the ROLE1 system. The casualties transferred to ROLE1 are unloaded and triaged. The P1 and P2 casualties are gathered at the *Preparation Post* stock and await treatment in the *Casualty Ward* stock. Transfers to the *Casualty Wards* are enabled by available physicians and again, P1 is preferred to P2. Casualties after treatment wait at the *Transfer Post* for transport from ROLE1 which is out of the scope of the model.

## 5. Experiment Results and Discussion

This section is divided into two parts. The first describes the generated flows of casualties from the recorded events in the CAX and the second contains the results of the design of the experiment (DoE) based on the executions of the implemented model of ROLE1 structure and behavior in Vensim.

### 5.1. Casualties Generated by the Model Reusing CAX Recorded Data

The flows of events of two CAXs were used to generate the flow of casualties. The first deals with offensive activities of own troops and the second deals with the situation when own forces are defensive. This approach of two scenarios was selected to demonstrate the potential differences in total casualties between defense and offence activities and to create a sample of the dispersion of casualty types in both scenarios.

Both CAXs scenarios replicate actual operational data of the Czech Armed Forces, namely tank battalion force structures in offense and mechanized battalion force structures in defense. Both battalions have relatively similar structures and staffing. Both CAXs scenarios were run with the support of the OneSAF constructive simulation [18]. This is an entity-level constructive simulation with the capacity to simulate and visualize the movements of even a single soldier. However, when the units are in formation, meaning inside vehicles, which is most of the time, the effects on individuals are not counted. Therefore, there is no casualty directly counted for a single soldier. To overcome this issue, the events generated by OneSAF, related to vehicle damage, are translated into the flow of casualties implemented in the filter.

Event types generated by OneSAF on the vehicles that were identified and used are:

- Fire capacity damage (FCD), a Boolean type event describing the situation when a vehicle is/is not able to fire;
- Mobility capacity damage (MCD), a Boolean type event describing the situation when a vehicle is/is not able to move;
- Catastrophic damage (CD), a Boolean type event describing a situation when a vehicle is/is not totally destroyed.

Total battle casualties (TBC) are expressed as killed in action (KIA), plus wounded in action (WIA). WIA is divided into three categories of casualty types as P1, P2 or P3. Filters translate events to the casualty types and KIA as follows:

- P3 = FCD&!CD. The entire vehicle crew is lightly wounded if only an FCD event is recorded;
- P2 = MCD&!CD. The entire vehicle crew is medium wounded if only an MCD event is recorded;
- P1 = FCD&MCD!CD. The entire vehicle crew is seriously wounded if only both FCD and MCD events on the same vehicle are recorded;
- KIA = CD. The entire vehicle crew is killed if only a CD event is recorded.

Table 1 summarizes the total number of different types of casualties and KIA in both defense and offense scenarios, replayed and filtered, based on the previously applied principles.

The total number of casualties, P1 + P2 + P3, in both scenarios, is almost identical. However, there are differences in the total numbers, time distribution and position information of the casualty types P1, P2 and P3 in these two scenarios. As for the total number of KIA, it corresponds to the very theoretical expectation and practical experience from the current battlefield of higher fatalities in the defense operation than in the offense operation. An important result is the significantly higher number of P1 in the defensive than offensive operations.

### 5.2. Design of the Experiment for ROLE1 Structure Optimization

The study problem explained in Section 3 is framed around the optimization of the values of ROLE1′s structure parameters that satisfy the condition of having a maximum of two hours between when the casualty occurred on the battlefield and the casualty’s transportation to ROLE2.

The experimental setup for this study consists of independent, controlled and dependent variables. Independent variables describing the key ROLE1 structure parameters of the experiment are:

- The total number of physicians ($Physicians_{NUMBER}$);
- The total number of ambulances ($Ambulances_{NUMBER}$);
- The distance between ROLE0 and ROLE1, expressed as the total time needed to arrive at ROLE0 from ROLE1, to put casualty into Ambulance and bring them to ROLE1 ($t_{TOROLE1}$). This variable was deliberately designed as time one. The reasoning behind having a time instead of distance variable is its independence upon the terrain, the environmental conditions and the type of vehicle used for transportation.

The model of the ROLE1 structure and behavior, as described in Section 4.2, implemented in Vensim, is deterministic. Therefore, the controlled variables that are not varied throughout the experiment runs are kept constant and do not follow any distribution function. These variables’ values were gathered as the worst-case scenario estimates through the subject matter experts’ interviews and validated during a staff exercise replicating entire the ROLE1 functions. In the staff exercise, two single runs were executed, using the casualty flow generated from the model reusing the CAX recorded data.

It was proven that the ROLE1 function works within its given limits. However, the further examination and search for the independent variables’ variation was not possible because of the extreme resource demand. Therefore, the model of the ROLE1 structure and behavior and DoE was implemented.

The following list contains all the controlled variables in the experiment.

● The time of triage $t_{TRIAGE}=1\;\min$;
● Loading time per casualty $t_{LOAD}=3\;\min$;
● Unloading time per casualty $t_{UNLOAD}=2\;\min$; and
● the time of treatment $t_{TREATMENT}$;
○ P1 $t_{TREATMENT}=15\;\min$;
○ P2 $t_{TREATMENT}=10\;\min$;
○ P3 $t_{TREATMENT}=5\;\min$.

The only dependent variable of the experiment is the total time needed to treat the casualty from the moment they are wounded to the time they are ready for transport from ROLE1 to ROLE2 ($t_{TOTAL}$).

Therefore, the experiment looks for the combinations of values of independent variables $Physicians_{NUMBER}=\left\{2,3,4\right\}$, $Ambulances_{NUMBER}=\left\{2,3,4,5,6\right\}$ and $t_{TOROLE1}=\left\{10,15,20\right\}$ while $t_{TOTAL}$ is less or equal to 120 min in both offense and defense scenarios. Subject matter experts gave estimates of the ranges for all the independent variables.

The set of all the permissible solutions is defined as follows:

$$S_{P}=\{Physicians_{NUMBER}\}\times \{Ambulances_{NUMBER}\}\times \left\{t_{TOROLE1}\right\}$$

$$|(t_{TOTAL_{OFFENSE}}\leq 120\;min)\wedge \left(t_{TOTAL_{DEFENCE}}\leq 120\;min\right)$$

Let us define a simple cost function *F* evaluating the resources (physicians and ambulances) as

$$F\left(Physicians_{NUMBER},\;Ambulances_{NUMBER},\;t_{TOROLE1},\;Dataset\;\right)\;=Physicians_{NUMBER}+Ambulances_{NUMBER}$$

where *Dataset* is either the offense or defense set of input data.

Splitting the $S_{P}$ set into the three subsets $V_{i}$, i={10, 15, 20}, given by the value of $t_{TOROLE1},$ we can find for each value a permissible optimal solution $s_{OPTIMAL_{i}}$ (if it exists), minimizing the cost function F on a subset $V_{i}\subset S_{P}$ as

$$s_{OPTIMAL_{i}}=s|F\left(s\right)=\underset{q\in V_{i}}{\min}F\left(q\right)\;$$

The 3 × 5 × 3 factorial experimental design brought 45 runs for each offensive and defensive scenario. The results of the experiment are found in Table 2 and Table 3, where the green rows indicate the achieved condition of not exceeding 2 h total time ($t_{TOTAL}$), and the blue rows indicate the optimal solution for three subsets given by the value of $t_{TOROLE1}$ while treating the offense and defense scenarios individually. The condition of 2 h is applicable only if the limit is met for both the P1 $t_{TOTAL}$ and P2 $t_{TOTAL}$. The P3 $t_{TOTAL}$ was not measured in the experiment. As already explained, this casualty type does not create any additional load to the ROLE1 system, therefore it can be disregarded.

Experiment runs in the offense scenario demonstrate that the number of physicians and the number of ambulances in ROLE1 should not be less than three. An interesting finding is that the higher numbers of ambulances than the critical value, three, makes no significant difference and the same is applicable for the number of physicians.

Based on the results for the defense scenario, there should not be less than three physicians and two ambulances. However, following the results of run#1–run#5, when the ROLE 0 and ROLE1 are close to each other, even two ambulances and two physicians are sufficient for the total time below two hours. That is not the case for the offensive scenario. Interesting results came from the comparison of run#6 and run#7. Logically, the expected effect of the increased number of ambulances would be the decreased time for the P1 and P2 casualty types. However, because of the priority in the loading of P1 before P2, the added ambulance moved P1 faster and P2 had to wait longer. This leads to the conclusion that the prioritization can be a counter-productive to the overall objectives and should be reconsidered.

When combining these two sets of experimental results together to get the $s_{OPTIMAL_{i}}$ applicable for both the offense and defense scenarios, the recommended solution is in Table 4.

The overall recommendation is for the internal structure of ROLE1 to have three ambulances and three physicians for any kind of current operation and any distance between ROLE0 and ROLE1 within the limit of 20 min.

## 6. Conclusions

ROLE1 has the paramount importance in casualty reductions in the current operations representing a complex system. The execution of the 3 × 5 × 3 factorial experimental design for the model of ROLE1 structure and behavior, consuming the outputs of the model of battlefield casualties, brought the recommended structure of ROLE1, expressed in the values of the number of physicians and the number of ambulances related to the distance between ROLE1 and the current battlefield. The experiment results influence the content of a new national standard operating procedure for battlefield casualty treatment.

The secondary benefit of the developed model of ROLE1 structure and behavior comes from the fact that it may be used as an auxiliary planning tool for battalion commanders when deciding the geographical position of ROLE1 in relation to ROLE0. The deterministic model can evaluate the maximum time perimeter where the ROLE1 should be employed, assuring timely casualty treatment.

A constraint of the model of the ROLE1 structure and behavior comes from its nature being deterministic. Having more life experiments executed while studying the expected values of the current model parameters treated as the constant would enable building the probability distributions and creating a stochastic model. It would definitely increase the reliability of the study results. However, gathering experimental data on controlled variables, $t_{LOAD}$, $t_{UNLOAD},\;t_{TRIAGE}$ and $t_{TREATMENT}$ is a very resource-demanding activity. Therefore, the worst-case scenario estimates by subject matter experts were applied.

The study unique approach of reusing events recorded by the constructive simulation during the computer assisted exercises (CAXs) and filtering them into the time and position distributions of classified casualties forms a new methodology of generating casualties. The main advantage of the methodology is adding the human decisions through a training audience into the generated flow of casualties, which is not the case in the statistical approach and the closed-loop simulation approach to casualty estimation.

The constraint of the methodology is mainly founded in the limited number of already executed and recorded CAXs, which is caused by the enormous resource consumption for their preparation, execution and analysis. However, CAX in its essence brings the stochastic phenomena into the casualties’ generation process, because the constructive simulation supporting the CAX execution is fully stochastic; moreover, human factors are represented in the decision making by TA, HICON and LOCON.

The use of the military blended experiment term was inspired by the blended learning approach where two radically different learning methods are exercised to reach the single objective. We see the parallel in our study methodology when mixing two families of simulation-driven experimentation. The first one, the human-in-the-loop simulation experiment, represented by the process of casualty generation, employs human beings in the decision making. The second one, the closed-loop simulation experiment, represented by the model of the ROLE1 process, excludes the human factors and their influence on the model.

As for the way forwards, the authors will prepare live experimentation campaigns, gathering experimental data for altering the ROLE1 structure and behavior model into its stochastic form. There is a limited way to get field experimental data that can be reused for military studies, therefore further constructive simulations should be analyzed to reveal whether they may generate the flow of casualties directly from the recorded CAXs or through a designed innovative transformation mechanism that has been demonstrated in the study.

## Figures and Tables

**Table 1:** Summary of casualties in defense and offense scenarios generated by the model reusing the CAX recorded data.

Offense		Defense	
Casualty Type	Total Number	Casualty Type	Total Number
P1	10	P1	16
P2	20	P2	20
P3	19	P3	16
P1 + P2 + P3	49	P1 + P2 + P3	52
KIA	14	KIA	24

**Table 2:** Results of 45 runs of the factorial experiment in the offensive scenario.

Offense Scenario						
**Run#**	$Physicians_{NUMBER}$	$t_{TOROLE1\;}(\mathrm{Minutes})$	$Ambulances_{NUMBER}$	$P1\;t_{TOTAL}$ (Minutes)	$P2\;t_{TOTAL}(\mathrm{Minutes})$	*F* (–)
1	2	10	2	82	216	4
2	2	10	3	81	209	5
3	2	10	4	81	209	6
4	2	10	5	81	209	7
5	2	10	6	81	209	8
6	2	15	2	79	221	4
7	2	15	3	93	206	5
8	2	15	4	96	205	6
9	2	15	5	96	205	7
10	2	15	6	96	205	8
11	2	20	2	109	219	4
12	2	20	3	85	219	5
13	2	20	4	105	217	6
14	2	20	5	105	217	7
15	2	20	6	105	217	8
16	3	10	2	76	104	5
17	3	10	3	75	88	6
18	3	10	4	75	88	7
19	3	10	5	75	88	8
20	3	10	6	75	88	9
21	3	15	2	68	133	5
22	3	15	3	85	102	6
23	3	15	4	85	101	7
24	3	15	5	85	101	8
25	3	15	6	85	101	9
26	3	20	2	91	176	5
27	3	20	3	82	109	6
28	3	20	4	92	95	7
29	3	20	5	92	96	8
30	3	20	6	92	96	9
31	4	10	2	66	81	6
32	4	10	3	71	72	7
33	4	10	4	71	72	8
34	4	10	5	71	72	9
35	4	10	6	71	72	10
36	4	15	2	67	123	6
37	4	15	3	79	91	7
38	4	15	4	81	82	8
39	4	15	5	81	82	9
40	4	15	6	81	82	10
41	4	20	2	88	175	6
42	4	20	3	74	97	7
43	4	20	4	91	86	8
44	4	20	5	91	86	9
45	4	20	6	91	86	10

**Table 3:** Results of 45 runs of the factorial experiment in defensive scenario.

Defense Scenario						
Run#	$Physicians_{NUMBER}$	$t_{TOROLE1}\;(\mathrm{Minutes})$	$Ambulances_{NUMBER}$	$P1\;t_{TOTAL}$(Minutes)	$P2\;t_{TOTAL}$ (Minutes)	*F* (–)
1	2	10	2	88	120	4
2	2	10	3	89	97	5
3	2	10	4	78	113	6
4	2	10	5	78	113	7
5	2	10	6	78	113	8
6	2	15	2	104	108	4
7	2	15	3	99	130	5
8	2	15	4	99	130	6
9	2	15	5	83	126	7
10	2	15	6	83	126	8
11	2	20	2	96	147	4
12	2	20	3	88	129	5
13	2	20	4	93	124	6
14	2	20	5	93	124	7
15	2	20	6	93	124	8
16	3	10	2	79	94	5
17	3	10	3	82	81	6
18	3	10	4	63	73	7
19	3	10	5	63	73	8
20	3	10	6	63	73	9
21	3	15	2	86	89	5
22	3	15	3	78	89	6
23	3	15	4	78	89	7
24	3	15	5	68	89	8
25	3	15	6	68	89	9
26	3	20	2	89	120	5
27	3	20	3	82	109	6
28	3	20	4	92	95	7
29	3	20	5	92	96	8
30	3	20	6	92	96	9
31	4	10	2	67	82	6
32	4	10	3	71	79	7
33	4	10	4	63	70	8
34	4	10	5	63	70	9
35	4	10	6	63	70	10
36	4	15	2	86	87	6
37	4	15	3	78	93	7
38	4	15	4	78	93	8
39	4	15	5	68	77	9
40	4	15	6	68	77	10
41	4	20	2	89	117	6
42	4	20	3	73	88	7
43	4	20	4	78	87	8
44	4	20	5	78	87	9
45	4	20	6	78	87	10

**Table 4:** ROLE1 structure parameters optimized for the three permissible solutions subsets given by the value of $t_{TOROLE1}$

Run#	$Physicians_{NUMBER}$	$t_{TOROLE1}(\mathrm{Minutes})$	$Ambulances_{NUMBER}$	*F* (–)
16	3	10	2	5
22	3	15	3	6
27	3	20	3	6
